# A Daily Oscillation in the Fundamental Frequency and Amplitude of Harmonic Syllables of Zebra Finch Song

**DOI:** 10.1371/journal.pone.0082327

**Published:** 2013-12-02

**Authors:** William E. Wood, Peter J. Osseward, Thomas K. Roseberry, David J. Perkel

**Affiliations:** 1 Graduate Program in Neurobiology & Behavior, University of Washington, Seattle, Washington, United States of America; 2 Department Biology, University of Washington, Seattle, Washington, United States of America; 3 Department Otolaryngology, University of Washington, Seattle, Washington, United States of America; UCLA, United States of America

## Abstract

Complex motor skills are more difficult to perform at certain points in the day (for example, shortly after waking), but the daily trajectory of motor-skill error is more difficult to predict. By undertaking a quantitative analysis of the fundamental frequency (FF) and amplitude of hundreds of zebra finch syllables per animal per day, we find that zebra finch song follows a previously undescribed daily oscillation. The FF and amplitude of harmonic syllables rises across the morning, reaching a peak near mid-day, and then falls again in the late afternoon until sleep. This oscillation, although somewhat variable, is consistent across days and across animals and does not require serotonin, as animals with serotonergic lesions maintained daily oscillations. We hypothesize that this oscillation is driven by underlying physiological factors which could be shared with other taxa. Song production in zebra finches is a model system for studying complex learned behavior because of the ease of gathering comprehensive behavioral data and the tractability of the underlying neural circuitry. The daily oscillation that we describe promises to reveal new insights into how time of day affects the ability to accomplish a variety of complex learned motor skills.

## Introduction

Many complex motor skills in humans show subtle yet definite daily oscillations in performance (for a review see [1]). Early work focused on when the best time of day to teach might be [2]. Decades later careful studies involving motor tasks with minimal cognitive loads, such as sorting a deck of cards [3], revealed a correlation of both speed and accuracy with time of day, as well as body temperature. More recently, studies have found some success correlating complex sports performance in humans with time of day and body temperature [4,5]. For more complicated tasks, however, particularly those involving multiple participants, daily patterns become more difficult to observe. Alternatively, studying daily changes in motor performance in animal models with more tractable underlying neural circuitry may allow for progress toward understanding their mechanistic basis, which can then be related to human physiology.

Songbirds such as the zebra finch produce songs, which are complex, learned vocalizations. Their songs are learned through a trial-and-error process with auditory feedback over tens of thousands of repetitions before achieving adult song quality [6]. The male zebra finch learns to sing between the first and third month of life, after which period an individual bird sings the same stereotyped song, with very little evident variation, for the rest of his life [7]. Song consists of a stereotyped sequence of specific acoustic syllables, known as a motif. Adult male zebra finches continue to sing thousands of repetitions of their song motif per day, even in isolation and without external stimuli, and thus provide an excellent system for studying daily oscillations in a complex motor task.

We have undertaken a detailed analysis of the fundamental frequency (FF) of hundreds (often over one thousand) of zebra finch syllable renditions per animal per day. We focused on syllables with harmonic stack acoustic structure, allowing for accurate measurements of FF, and found a daily rhythm in the FF of harmonic syllables. As there is a growing body of evidence that changes in amplitude can mediate similar subtle changes in FF [8] we wondered if syllable amplitude followed a similar pattern and found that it does. We hypothesize that these oscillations are driven by underlying physiological factors shared across the animal kingdom. One possible mediator of the daily rhythm is the monoaminergic neuromodulator, serotonin (5-HT), which is involved in sleep and other circadian behaviors [9]. We tested whether lesions of the serotonergic system disrupt the daily oscillation in FF by re-analyzing data from a previous study [10], and we rule 5-HT out as a causal factor. Song production in zebra finches is a valuable model system for studying complex learned behavior, and the daily oscillation described here thus promises to reveal new insights into how time of day influences our ability to accomplish complex learned motor tasks.

## Materials and Methods

Ethics statement: All experimental procedures involving animals were approved by the University of Washington Institutional Animal Care and Use Committee (Protocol 3304-01).

### Animals

Adult male zebra finches were obtained from a commercial supplier or reared in our colony. Animals were housed in groups of 4-10 males, on a 13:11 hr light:dark cycle, with food and water available *ad libitum*. Cages were approximately 38x38x35 cm, with the microphone immediately outside the cage. All animals were part of a previous study investigating the actions of 5-HT in the robust nucleus of the arcopallium (RA), a premotor nucleus for song production [10].

### Song Analysis

Birds were individually housed in sound attenuation chambers (Acoustic Systems) at least seven days before and 14 days after lesion of the serotonergic system. We continually recorded spontaneous (undirected) vocalizations using Sound Analysis Pro (SAP) software [11]. Songs were sorted and analyzed using custom Matlab software and SAP. Zebra finch songs are highly stereotyped, making them especially well suited for in-depth analysis. The acoustic structure of song is arranged in a hierarchy, with 25-250 ms vocal units known as syllables strung together in a stereotyped sequence called a motif. Each song consists of one or several motifs, preceded by introductory notes and separated from each other by <100 ms of silence. We used custom Matlab software [12] to sort individual motifs from calls and cage noise, and all songs were high-pass filtered at 100 Hz using the Matlab ‘filtfilt’ function. Briefly, the program detected putative motifs based on peaks in the cross-correlation between the spectrogram of the song and that of a clean preselected motif. Such putative motifs were then sorted into complete motifs and partial motifs (which were discarded) based on their spectral similarity with the preselected clean motif using thresholds set by the experimenter. For motifs for which such analysis did not allow unambiguous distinction, an additional principal components analysis (PCA) of the spectrograms of putative motifs allowed us to sort motifs from other sounds. This analysis allowed us to successfully sort >90% of the motifs sung by a bird on a given day (assessed by comparing hand sorting with the automated sorting by the program). The <10% which were discarded generally represented motifs with abnormal syllable order or containing abnormal syllables. Once clean motifs had been sorted, individual syllables were sorted into catagories using custom Matlab software somewhat similar to previously reported methods [13]. Briefly, we ran clean motifs through the SAP batch processing module to separate individual syllables and calculate spectral and temporal features. These data were imported into Matlab and sorted by a semi-automated procedure in which the user selects spectral and temporal feature values for each syllable (as well as the order in which they occur). Using these methods we sorted and analyzed the majority of syllables produced daily by each animal over the course of the experiment.

### Calculation of Fundamental Frequency and Amplitude

A large number of song spectrograms were visually analyzed to determine an optimal window size of 23-ms from which to calculate the fundamental frequency of multiple syllables and birds. A spectrally stable and harmonic portion of the syllable, as determined visually by having no obvious deviations or sloping profile, was windowed at a constant time point from the onset of the syllable. The power spectrum of a segment of the syllable waveform was calculated. The fundamental frequency of the syllable was calculated from the frequency bin of greatest spectral power using a weighted average from the four neighboring bins and the inter-harmonic frequency-based methods previously employed in the field [14]. One to three syllables were analyzed per bird- we tried to analyze any syllable that had harmonic portions of significant length.

Amplitude was calculated using the SAP batch processing module. A baseline amplitude was not calibrated; the SAP default baseline of 70 dB was used for amplitude calculations. In using the (arbitrarily chosen) SAP baseline, amplitude calculations reflect the relative intensity of syllable renditions to each other, rather than their absolute intensity. Amplitude measurements will depend on the position of the bird relative to the microphone and substantial variation may be expected. 

### Calculation of Temporal Changes of Syllable Features

The daily time course of the mean fundamental frequency and amplitude of each syllable was calculated using the MATLAB smoothing curve function rloess, a non-parametric regression method related to loess regression, which decreases the weighting of outlying data points allowing better curve fits when there are long gaps between data points (for example when there is an hour of no singing in the day). Rloess points were calculated at 14 time points spaced evenly through the day beginning with the first motif of the day and ending with the last. Rloess parameters alpha and beta were set to 0.7 and 2 respectively and were determined empirically to reflect the best fit while minimizing over-smoothing.

The average morning and evening derivative for each day were calculated using the rloess curve values in the first and last three hours of light, respectively, divided by the time between points. For example if the FF in the first hour was 550Hz and the FF in the third hour was 570Hz, and there were 2 hr between points, the derivative would be (570Hz-550Hz)/2hr = 10 Hz/hr.

Singing rate was calculated by creating 13 bins from the 14 time points (described in the rloess calculation) evenly spaced through the day. For each bin, the number of syllable renditions sung, the average FF of those syllable renditions, as well as the average ampltidue of the syllable renditions was calculated.

The difference from mean FF of a period was defined as the difference between the daily average FF and the FF of the first hour of that period. Sporadic singing occasionally resulted in erratic derivative values, and values greater than three standard deviations from the mean were excluded.

The FF residual value for each syllable rendition was calculated by subtracting a corresponding linearly interpolated rloess curve value from the FF of the syllable rendition. For example, for a syllable rendition sung at two hours and seventeen minutes after the beginning of lights on, a theoretical FF value would be calculated by linearly interpolating between the second hour and third hour rloess points to give a theoretical FF value exactly at two hours and seventeen minutes after lights on. This theoretical FF value was subtracted from the FF of the actual syllable rendition, giving the residual. 

Morning and evening amplitude derivatives, the difference from mean amplitude, and amplitude residuals for syllable renditions were calculated in the same manner as described for FF above.

### Surgery

See [10] for a comprehensive treatment of the 5-HT lesion and effects. Briefly, FF of syllables was transiently lowered following 5-HT lesion [10], and while 5-HT levels did not recover, FF did. We injected 500 nl of 5,7-dihydroxytryptamine creatinine sulfate salt (5,7-DHT, Sigma-Aldrich) into the third ventricle (coordinates: 0 lateral, -1 rostral, 3.75 deep, 40° head angle). While 5,7-DHT is a neurotoxin mostly selective for serotonergic terminals [15], dopaminergic neurotoxicity was blocked by pretreatment with 0.1 ml desipramine hydrochloride (2 mg/ml, Sigma-Aldrich) given by IM injection. Two weeks after injection of either 5,7-DHT (n = 5) or saline (n = 6), animals were sacrificed by decapitation during isoflurane anesthesia between 9 AM and 12 PM. Brains were sliced at 300 μm using a vibrating microtome (Oxford) and RA, adjacent arcopallium, the song nucleus HVC (proper name ; Reiner et al., 2004), adjacent nidopallium, Area X, striatum, cerebellum, and brain stem were dissected and stored at -80°C. Lesion effectiveness and specificity were verified by high pressure liquid chromatography (HPLC, Vanderbilt University Core Facility) for a variety of monoamines in these tissue punches.

### Definitions of Baseline, Lesion, Recovery

Baseline for all birds was established using the average of three stable days in the one to five days before surgery. After surgery, birds often sang little or not at all for a few days, and thus we excluded days following surgery when birds sang fewer than 30% of the pre-surgery average number of songs (generally 2-5 days were excluded). The lesion period was defined as the first three days following resumption of singing. Recovery was defined as 7-10 days following resumption of singing, unless stated otherwise.

### Statistics

Values presented are mean ± SEM. Unless otherwise mentioned, p values represent results from unpaired student’s t tests.

## Results

### Daily Patterns in Song

**Figure 1:**
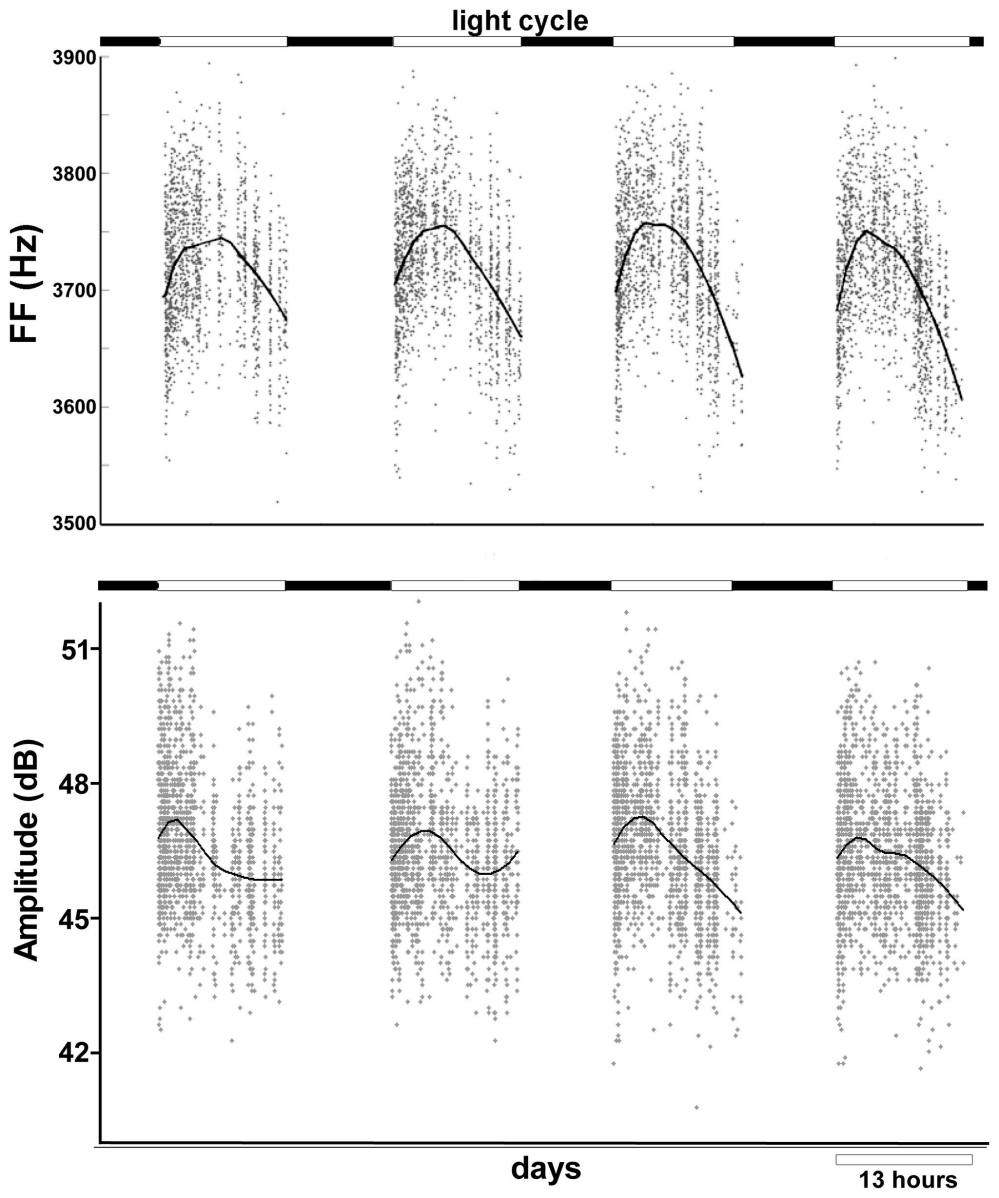
Syllable FF and amplitude follow a daily oscillation. **A**) The FF for 1 syllable across 4 days of singing, each point represents one rendition of the syllable. The black lines are RLOESS fits. **B**) The amplitude for the same syllable and same renditions as in A.

**Figure 2:**
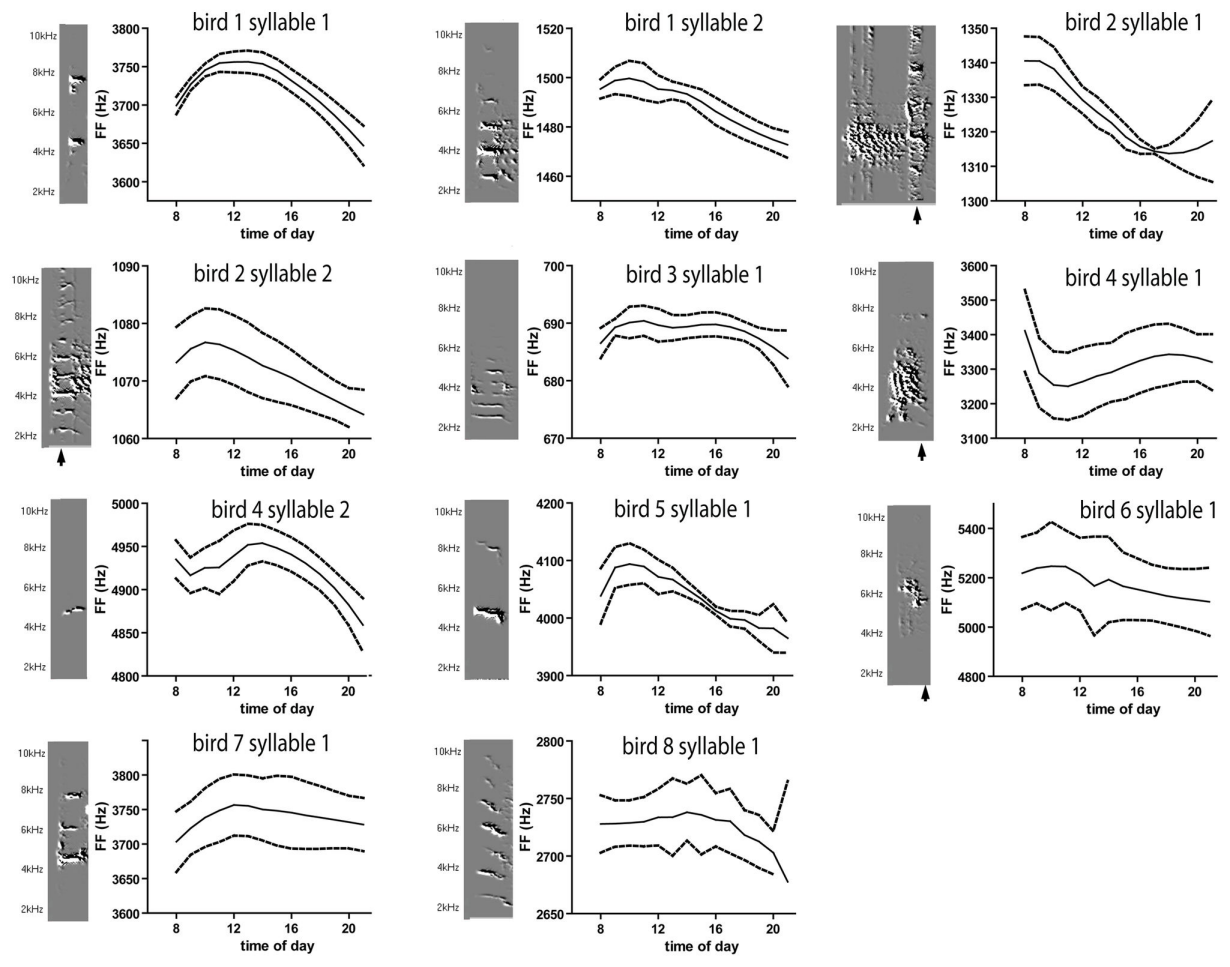
FF of harmonic stacks, and of harmonic subsyllabic elements, follows a daily oscillation, albeit with substantial variation and noise. Averaged daily RLOESS fits of FF (± 1 SD) for 11 syllables from 8 animals. Average number of days = 13.25 ± 7.025.

**Figure 3:**
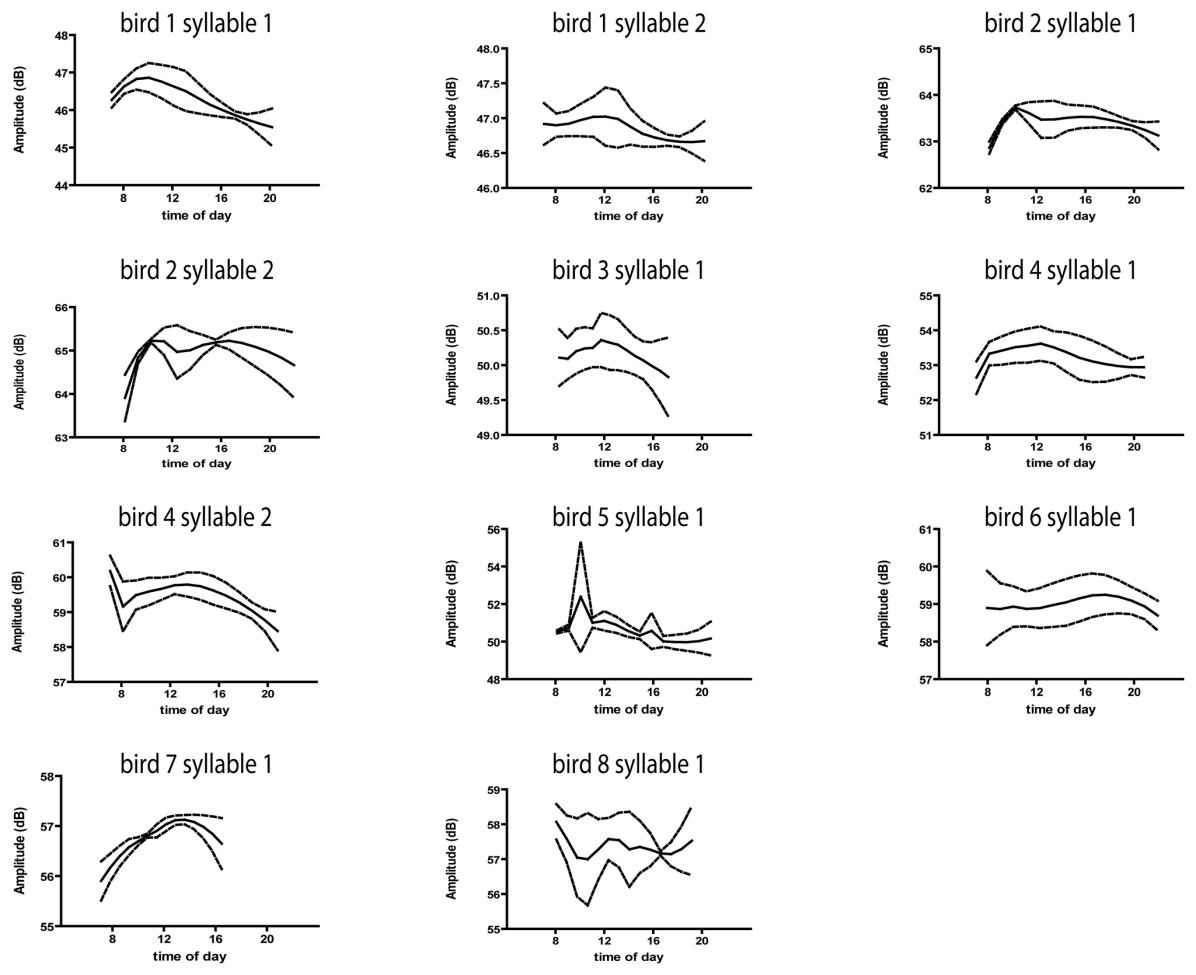
Amplitude of harmonic stacks, and of harmonic subsyllabic elements, follows a daily oscillation, albeit with substantial variation and noise. Averaged daily RLOESS fits of amplitude (± 1 SD) for 11 syllables from 8 animals. Average number of days = 4.82 ± 0.60.

**Figure 4:**
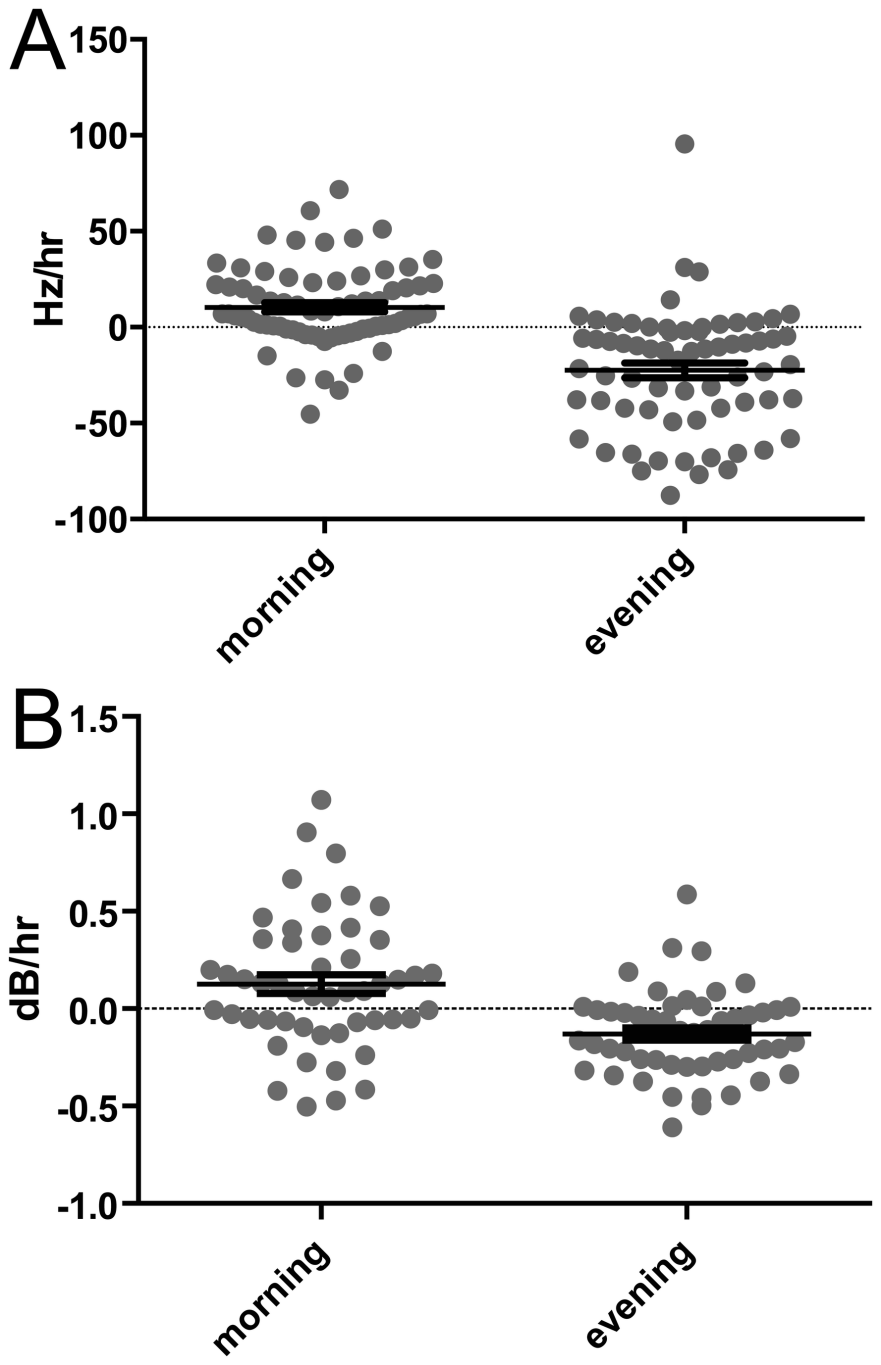
Across animals and across harmonic syllables (and harmonic subsyllabic elements) both FF and amplitude tend to rise in the morning and fall in the evening. Plotted are the slopes of the RLOESS fits for the first three hours of the day and the last three hours of the evening- each point represents the slope of 1 syllable from 1 animal from one morning or evening. Note the apparent ‘v’ shape of the data is an artifact of the algorithm used to make all symbols visible on the graph and does not connote information.

We found that FF and amplitude of harmonic syllables followed a daily pattern (Figures 1-4). As seen for an example bird examined over four consecutive days (Figure 1) FF and amplitude were quite variable from rendition to rendition (individual symbols), but tended to rise over the course of the morning and fall during the evening (black curves).

These tendencies were seen in most of the 11 syllables examined from 8 birds (Figures 2 and 3). Figure 2 illustrates the rloess fit of all FF values for each syllable averaged across multiple days. Figure 3 illustrates the average rloess fit of all amplitide values for each syllable averaged across multiple days. For most syllables, both FF and amplitude showed a daily oscillation.

We quantified this oscillation by measuring the slope of FF or amplitude for the first 3 hours of the day or last 3 hours of the evening. The morning FF slope was 10.35 ± 2.407 Hz/hr (Figure 4A ; p < 0.0001) and the morning amplitude slope was 2.990 ± 1.144 dB/hr (Figure 4B; p = 0.0118). FF slope in last three hours of the evening was -22.46 ± 3.857 Hz/hr (Figure 4A; p < 0.0001) and evening amplitude slope was -3.152 ± 0.7639 dB/hr (Figure 4B; p = 0.0001).

Each point in Figure 4 represents one syllable examined on one day. If we average all syllables and days for each individual bird, the morning slope had a nearly significant positive trend (8.5 ± 3.7 Hz/hr ; p = 0.054) and the evening slope was significantly negative (-26.0 ± 6.6 Hz/hr ; p = 0.0058). The same analysis for amplitude resulted in a morning slope with a positive trend (5.0 ± 3.5 dB/hr ; p = 0.2005) and a evening slope which was significantly negative (-3.1 ± 1.1 ; p = 0.0273). This pattern was also evident via the rloess smoothing curve (Figures 1-4). Fundamental frequency started slightly lower than average in the morning, while morning amplitude was not significantly different from average (-9.010 ± 3.974 Hz, p = 0.0261 for FF ; -0.06256 ± 0.08478, p = 0.4639 for amplitude). 

We did not observe a tendency for amplitude or FF to correlate with the singing rate. For each syllable, we tested whether the number of syllable renditions sung during a binned unit of time was correlated with the average FF or amplitude of those syllable renditions. For each syllable, we calculated an R^2^ value, reflecting how well correlated amplitude or FF was to singing rate. For FF, only 4 syllables of the 11 syllables showed a significant correlation with singing rate; for amplitude, there were only 2 significant correlations. Additionally, the amount of correlation between amplitude or FF with singing rate was low. Averaging the R^2^ values (correlating amplitude or FF to singing rate) of the 11 syllables gave a mean FF R^2^ value of 0.09263±0.03246 and a mean amplitude R^2^ value of 0.03762±0.01267.

### 5-HT Is Not Necessary

**Figure 5:**
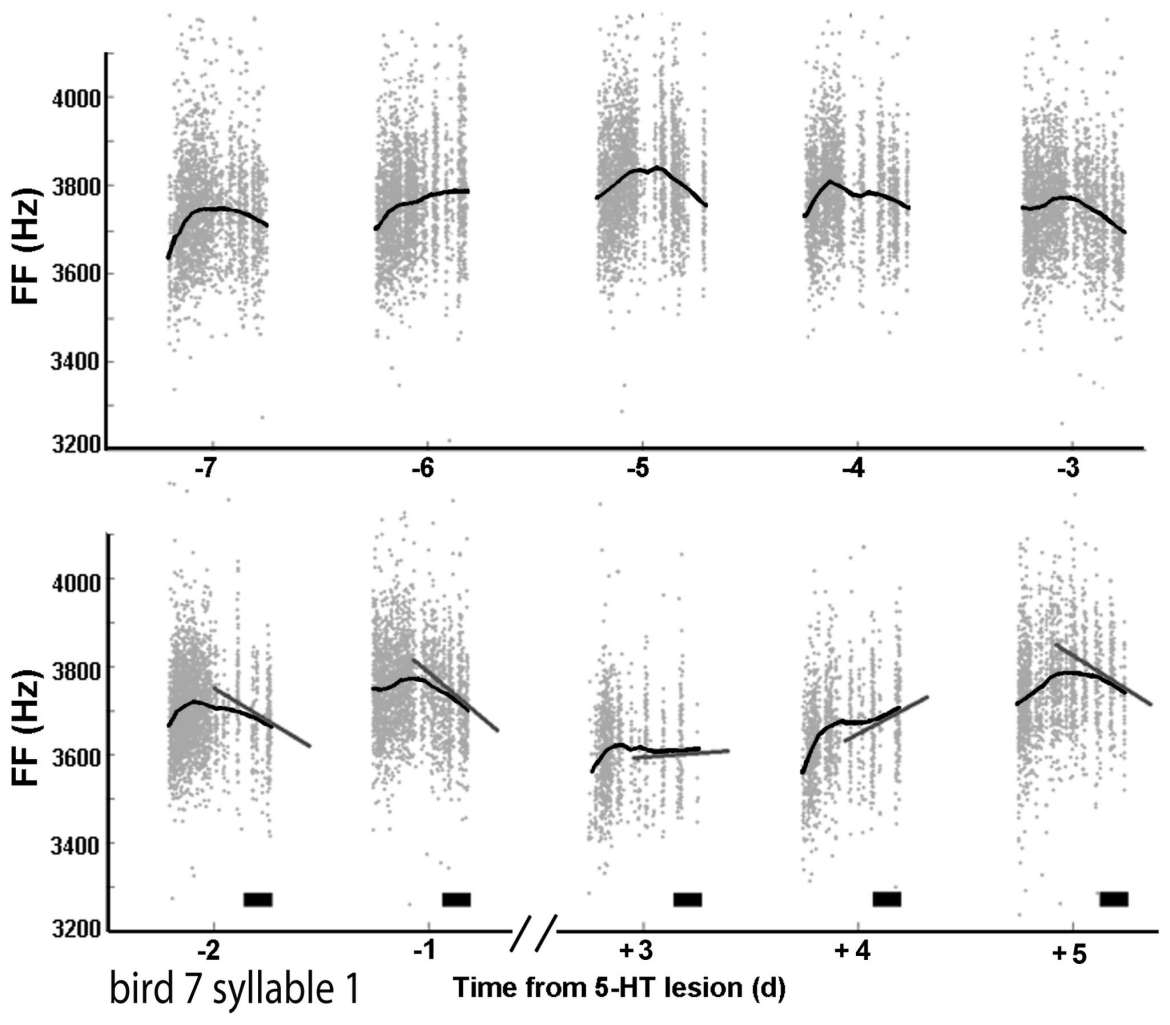
Daily patterns of FF in a typical harmonic syllable across seven days of baseline and three days post 5-HT lesion (after the bird began singing again). The black line is the RLOESS fit. The grey line is a line through the RLOESS fit during the last 3 hours of lights on, marked by the black bars at the base. Serotonin lesion disrupted the evening fall, and in fact FF instead rose in the evening on Lesion+3 day. Also visible on this graph is the overall decrease in FF following lesion (all points shifted downward after lesion). See Figure 7 for group data.

**Figure 6:**
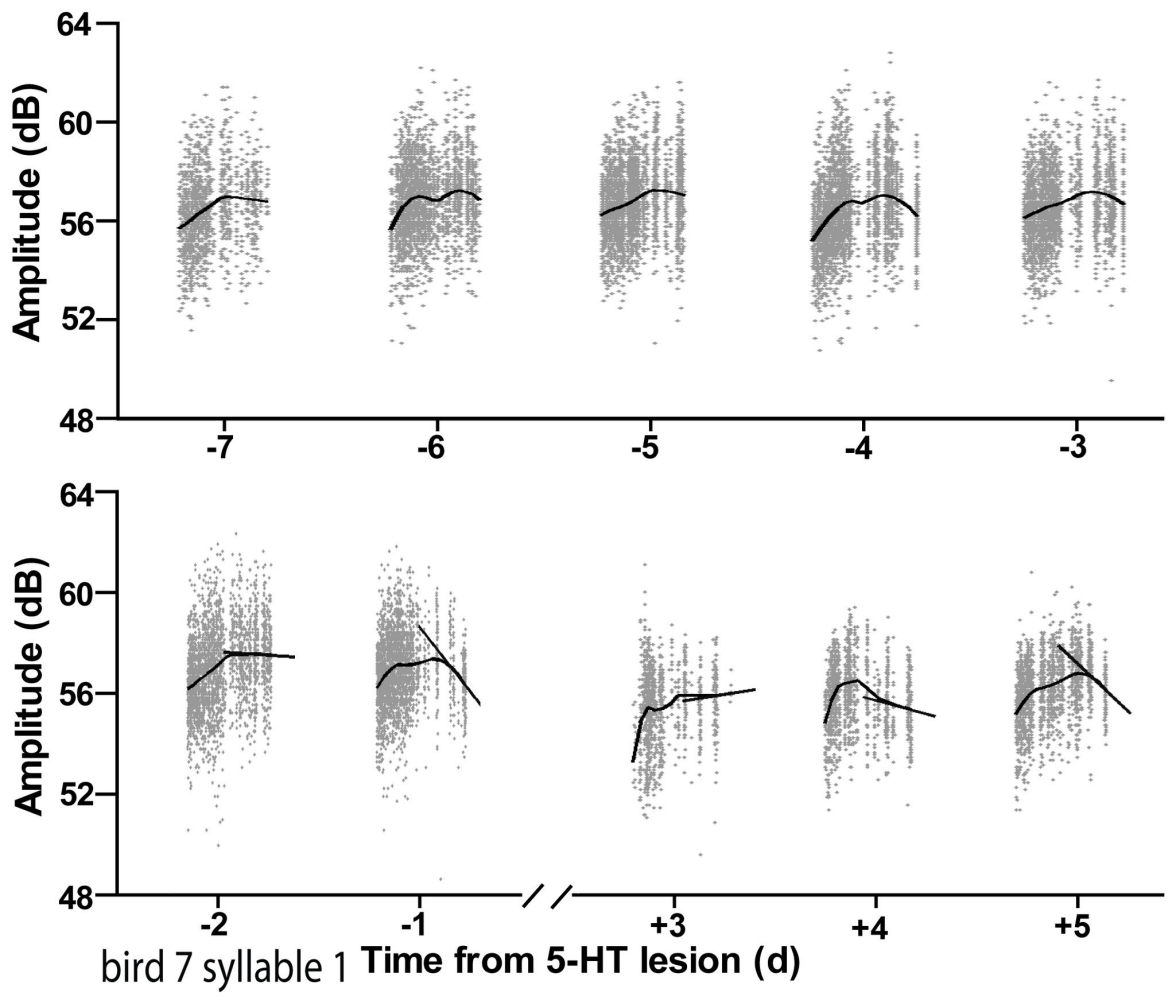
Daily patterns of amplitude in a typical harmonic syllable across seven days of baseline and three days post 5-HT lesion (after the bird began singing again). The black line is the RLOESS fit. The grey line is a line through the RLOESS fit during the last 3 hours of lights on, as in Figure 5. Serotonin lesion disrupted the evening fall, and in fact FF instead rose in the evening on Lesion+3 day. See Figure 7 for group data.

**Figure 7:**
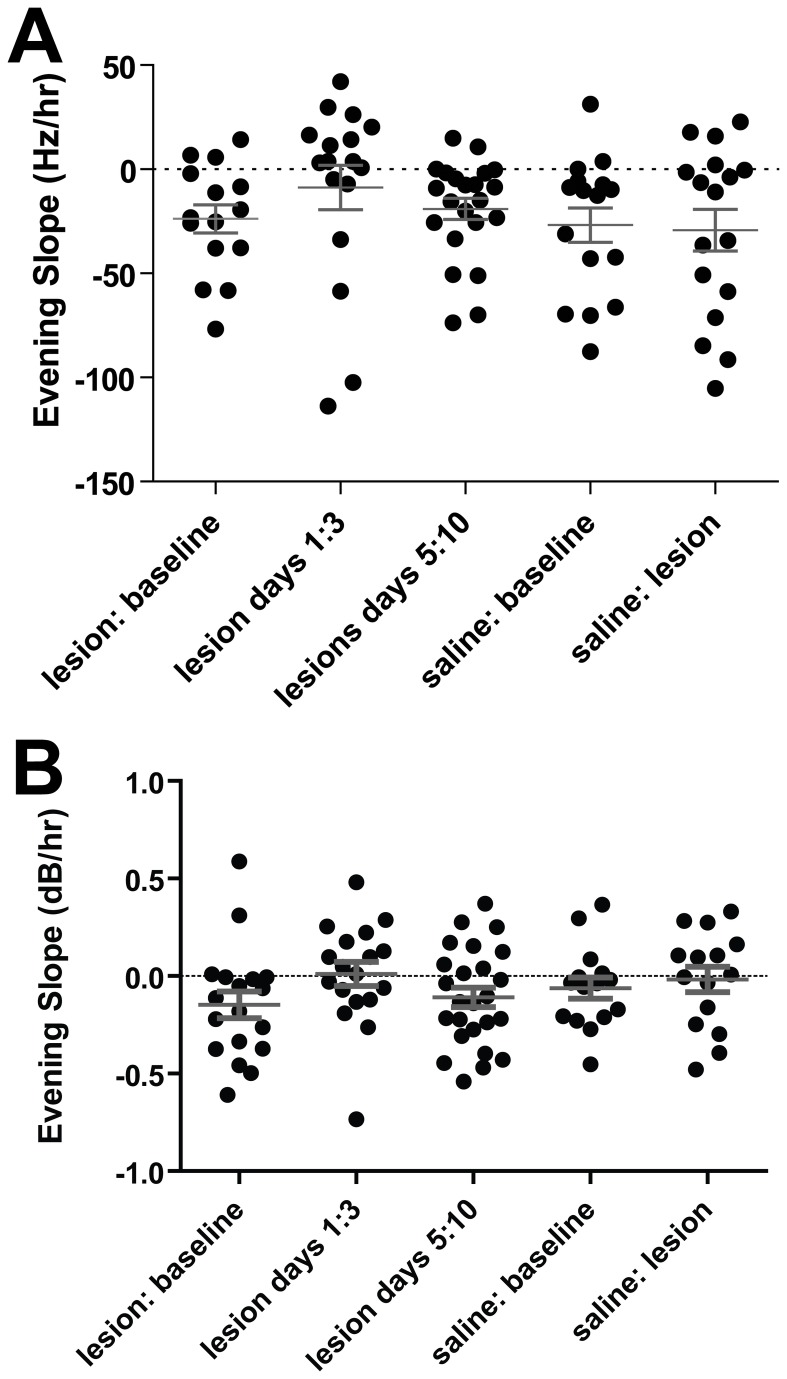
Normal 5-HT levels are not necessary for daily oscillation in FF or amplitude. Each symbol represents the slope of the RLOESS curve of FF or amplitude of one syllable from one animal on one day. **A**) There was a trend for evening slope of FF to become positive immediately following 5-HT lesion (while song was recovering but 5-HT was still depleted, p=0.0582 when compared to baseline slope). Oscillations were indistinguishable from baseline condition by 5 days after lesion surgery, at which point 5-HT levels continued to be drastically reduced. Saline controls showed no similar effects. **B**) Evening slope of amplitude increased significantly after 5-HT lesion (p=0.0075 when compared to baseline slope). Similarly to FF, the trend of the evening slope of amplitude to become positive ceased to persist 5 days after lesion surgery.

To ascertain whether 5-HT is necessary for the oscillation we lesioned the serotonergic system via injection of 5,7-DHT (n = 5) or saline (n = 6) into the third ventricle [10]. As reported, 5-HT levels were decreased to 1/3 the level of controls (p=0.0009), yet 5-HT lesion left song substantially intact. Following 5-HT lesion, the FF of harmonic song syllables was significantly reduced (Δ – 72.5 Hz ± - 36.6 Hz drop in lesion group, p=0.035 vs Δ 0.4 Hz ± 6.2 Hz in control animals, p > 0.05). Following the initial decline, FF recovered to baseline with a smooth trajectory over 3-5 days (Figure 5, recovery of 11.03 ± 2.102 Hz/day). Five days after lesion (when 5-HT levels were still drastically reduced) daily oscillations had entirely recovered to baseline, indicating that 5-HT is not necessary for the observed daily oscillation in FF or amplitude (Figures 6, 7).

Unlike FF, there was no significant reduction in amplitude following 5-HT lesion (p=0.3522, Δ-2.361 ± 2.420 dB drop in lesion group vs Δ 0.3296 ± 3.325 dB in control animals). The daily *oscillation* in amplitude, however, was transiently disrupted following 5-HT lesion but recovered to baseline on a similar timescale as the FF (Figure 6). 

In the days immediately following lesion (‘lesion period’) the evening portion of both oscillations was disrupted such that neither FF nor amplitude exhibited the usual evening decrease. During this period the average FF recovered to baseline values, possibly via adult error-correction. Whereas during baseline conditions we observed a positive slope in FF on only 20% of evenings, after surgery (during the period when animals were correcting their songs, the lesion period) we observed a positive slope on 64.7% of evenings (p < 0.05). Average evening slope in FF during baseline was -23.83 ± 6.794 Hz/hr, whereas after lesion it trended toward an increase of -8.75 ± 10.71 Hz/hr (Figure 7A, p=0.0582). 

Similarly, only 16.7% of recording days had a positive slope in evening amplitude during baseline conditions. This proportion increased to 55.6% after 5-HT lesion (p < 0.05). Average evening slope in amplitude during baseline was -3.513 ± 1.192 dB/hr. After lesion it increased to 0.6557 ± 0.8207 dB/hr (Figure 7B, p=0.0075). 

As we reported previously [10], 5-HT lesion did not alter the overall pattern of song production. The fraction of songs sung in the morning vs. the evening was unchanged, and there was no change in the total number of songs sung/day.

As reported previously, we generally did not see a change in spectral entropy following 5-HT lesion [10]. There was a transient increase in goodness of pitch following lesion, although we do not see any evidence of a daily oscillation in that or in any other song feature, and we do not report on them more here.

### Correlation of Slope Values with Morning Deviation from Average FF and Amplitude

**Figure 8:**
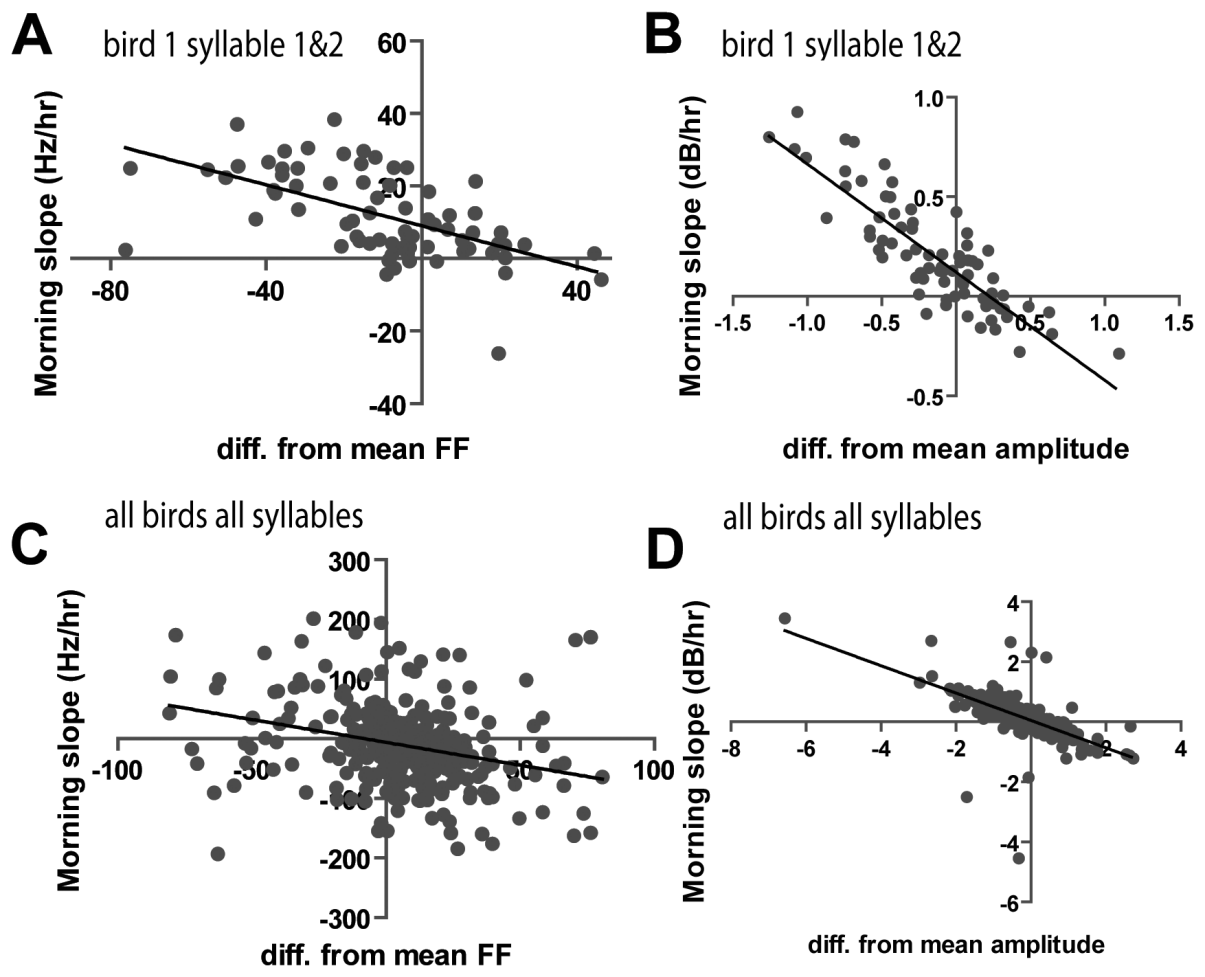
There is a correlation between morning difference from mean FF and initial trajectory of FF (slope of RLOESS fit). **A**, **B**) One example animal. Each symbol represents one syllable measured on one day. **C**,**D**) All syllables from all animals on all days- each symbol represents one syllable from one day.

We noted a correlation between the absolute FF in the first hour of the day and the subsequent slope of the oscillation across the morning. We therefore plotted the morning slope of FF against the difference between the starting FF and the bird's average FF over at least a week period. Morning slope was found to be significantly negatively correlated with difference in FF from weekly mean FF (Figure 8A, C). Thus, if the syllable started with lower FF than average in the morning, the slope of FF rise tended to be steeper than average (Figure 8C, p<0.0001, slope=-0.3765 Hz/hr, R^2^=0.404). No such relationship was found for slope of FF in the evening (p=0.109, slope=-0.1524 Hz/hr, R^2^=0.035, data not shown). A similar correlation was found for amplitude. Syllables with lower amplitude than average in the morning tended to have steeper amplitude slopes (Figure 8B, D p< 0.0001, slope=-13.80 dB/hr, R^2^=0.4504). Once again, no such relationship was found in the evening (p=0.3744, slope=-2.850dB/hr, R^2^=0.001848).

### Correlation between Amplitude and FF

**Figure 9:**
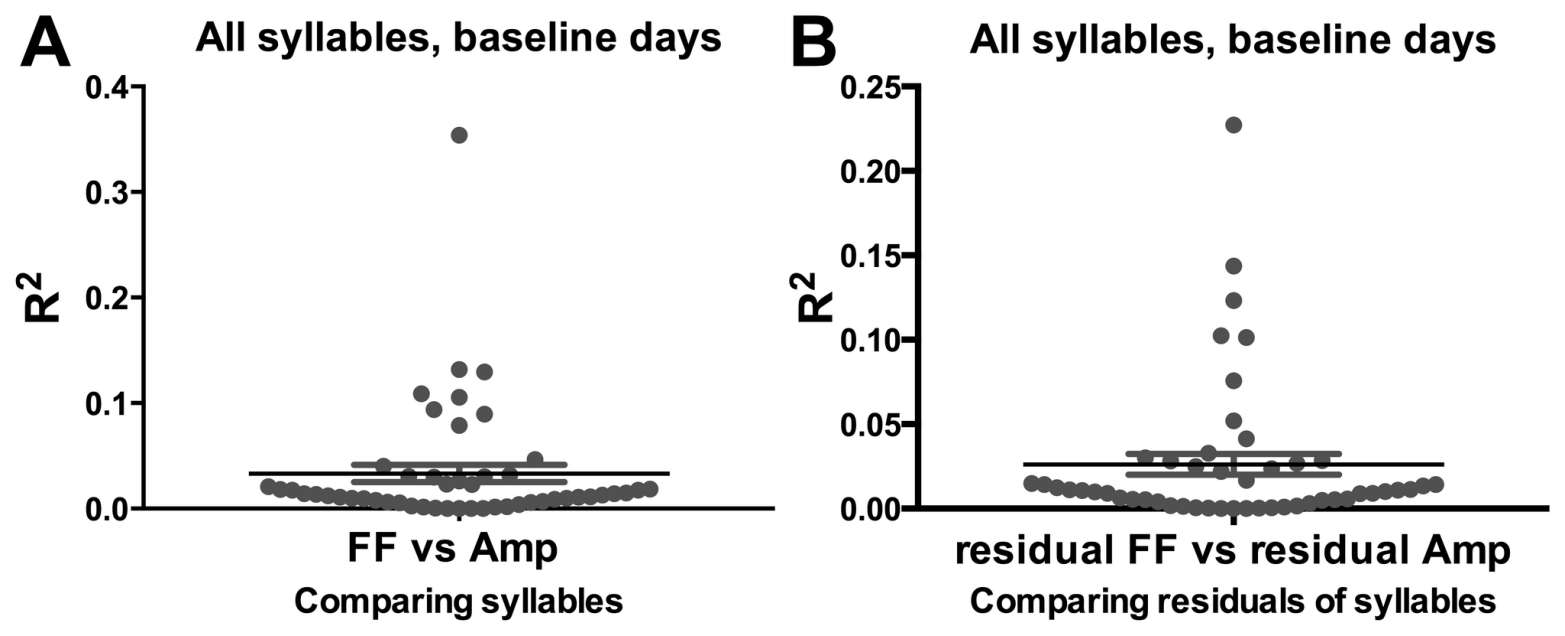
Syllable FF and amplitude are not correlated on a rendition to rendition basis. **A**) Each symbol represents the R^2^ value for the correlation of FF and amplitude of each syllable rendition sung by 1 animal for 1 day. **B**) Each symbol represents a R^2^ value for the correlation of FF residual and amplitude residual of each syllable rendition sung by 1 animal for 1 day.

Very little correlation between FF and amplitude was found on the level of individual renditions of syllables. For a given day, we calculated the R^2^ value, reflecting the degree of correlation of FF and amplitude across all renditions of a syllable for that day. Pooling these R^2^ values across animals and across days reflected a very low rendition-to-rendition correlation of FF and amplitude (Figure 9A ; mean R^2^= 0.03323 ± 0.008160).

In further support of the lack of correlation between FF and amplitude on the level of individual syllable renditions, the correlation between the FF residual and amplitude residual of each syllable rendition showed a low correlation as well. Each day once again produced a single R^2^ value, reflecting how correlated the FF and amplitude residuals of each syllable rendition were for that day. Pooling these R^2^ values across animals and across days reflected a very low correlation of FF and amplitude residuals (Figure 9B ; mean R^2^= 0.02618 ± 0.006144).

We did, however, observe a strong trend towards correlation between FF and amplitude rloess smoothing curve values across the day. This analysis eliminated the high rendition-to-rendition variability and allowed for a better comparison of the daily patterns in FF and amplitude. For each syllable examined, the FF and amplitude rloess smoothing curve values were correlated across all baseline days, resulting in an R value for each syllable. Of the 11 syllables, 7 had a significant positive correlation, 2 had no statistically significant correlation, and 2 had a negative correlation (R values were used instead of R^2^ due to the 2 negative correlations). Overall, the mean R value across all syllables tended to be positive (mean R value= 0.2837 ± 0.1311 ; p = 0.0558).

## Discussion

We report a daily oscillation in both FF and amplitude of harmonic syllables in the undirected songs of adult male zebra finches. This oscillation was embedded in substantial rendition-to-rendition variability, but was nonetheless statistically significant in all animals studied. FF and amplitude rise in the morning to a mid-day peak and then descend in the evening (Figures 1-4). This oscillation has not been reported before, likely because it is a subtle oscillation and obscured by trial to trial variation, requiring hundreds of syllables to be carefully analyzed per day before becoming clear. 

It is possible that the physical cause of the oscillations in both FF and in amplitude is an oscillation in air sac pressure. Air sac pressure is directly related with syllable amplitude, but the strength of this relationship has only recently become apparent [16,17] The most relevant work to our study is an elegant study by Amador and Margoliash [8] in which they rapidly (<100 ms) reduced air sac pressure during parts of harmonic syllables in the zebra finch and observed a direct and mostly linear decrease in FF as a result. The possibility that both oscillations we observed here are due to changes in air sac pressure is appealing in its parsimony but is difficult to prove from our data. We did not take air sac pressure measurements, and measures of syllable amplitude vary somewhat markedly depending on the direction of the beak relative to the microphone (indeed, we did not begin our study with the plan of measuring syllable amplitudes). It is perhaps surprising, then, that the oscillation in amplitude was observable at all. Given more accurate amplitude measurements (or ideally direct measures of air sac pressure) we would expect the amplitude oscillation to be even more pronounced. 

If oscillations in amplitude do in fact drive the oscillations in FF we would expect a very strong correlation between the measures on a rendition to rendition basis, and while we do see evidence of covariation, in most cases it is essentially nonexistent. (Figure 9). We are unable to determine to what extent this is due to errors in accurately measuring syllable amplitude and to what extent these two variables indeed behave independently. We find the hypothesis that the oscillation in FF is a result of an oscillation in air sac pressure to be compelling, but this will require air sac pressure measurements carried out over the course of multiple days for a definitive answer. 

### Candidate Mechanisms

Regardless of whether changes in air sac pressure are the proximate mechanism, there remains the question of what drives the proximate mechanism, whatever it may be. An intuitive explanation might be that motivation to sing leads to increased amplitude and FF. This explanation is unlikely to account for the observed oscillations, however, as during times when birds singing the most (the morning) amplitude and FF are low. Similarly, amplitude and FF peak around mid-day and fall in the evening, neither of which aligns with motivation to sing. 

An alternative explanation for the daily rhythm is body temperature. Manipulating the temperature of one song nucleus (HVC) alters the tempo of zebra finch song [18]. In addition, increases in song tempo associated with female-directed singing [19-21] involve increases in brain temperature that at least superficially resemble the period of oscillation in FF and amplitude observed here [22]. No studies to date have reported an effect of temperature on FF or amplitude. The tractable nature of the neural circuitry underlying song production in the zebra finch should greatly facilitate identifying the mechanisms underlying the daily rhythms in future studies. Temperature could also influence song via effects in the peripheral nervous system and muscles controlling respiration.

An additional factor that could contribute to the daily rhythm in song FF is circulating melatonin. Melatonin is a hormone that is a major coordinator of circadian rhythms in birds. Its receptors are heavily expressed in some song-system nuclei, including HVC and RA [23,24]. Exogenous and endogenous melatonin affects song tempo [24,25], but no reports have yet linked melatonin to changes in FF. Application of exogenous melatonin is reported to cause a small reduction in spontaneous firing rate in RA neurons. Because RA firing rate and FF are correlated [10,26] one can speculate that buildup of melatonin levels in the evening contributes to the steady decrease in FF that we observed here through a decrease in RA firing rate. Serotonin is a precursor to melatonin synthesis, however, so while we did not measure melatonin levels following 5-HT lesion it is very likely they were also significantly decreased. It is also entirely plausible that a circulating hormone, possibly melatonin, acts outside of the central nervous system, perhaps directly on musculature and thus alters air sac pressure and both amplitude and FF of syllables.

### Ruling out a role for 5-HT

Because 5-HT receptors are present in nuclei of the song system [27], and serotonergic lesions disrupt circadian rhythms in other systems [28], 5-HT was a plausible candidate neuromodulator to mediate this daily oscillation in FF. When we lesioned the serotonergic system, there was a transient loss of daily oscillations. Our finding, however, that the oscillation re-asserts itself within ~1 week after 5-HT lesion, despite strong and prolonged reduction in 5-HT level, argues strongly against this interpretation (Figures 4, 5). While we do not find it particularly likely, we cannot rule out the possibility that compensatory mechanisms (such as the upregulation of 5-HT receptors following lesion, [29]) allow for the birds to regain sufficient functioning of the serotonergic system to re-establish oscillations. In some cases, unmasking an effect of 5-HT on behavioral rhythms can require altering the photoperiod [30-32], and it is possible that such a manipulation will be required to observe an effect in songbirds.

### Adult Song Maintenance

It is possible that the observed oscillation in FF is the result of over-correction during the maintenance of FF of harmonic syllables [19,33-35]. Specifically, we hypothesize that there is some physiological pressure that causes FF to start low in the morning, reminiscent of the finding that juveniles sing more poorly immediately upon waking [36]. Possible causes include lower body temperature [1-3,22] or some consequence of sleep-related memory re-consolidation. The bird may begin correcting its song over the course of the morning until the desired FF is reached. If this were true we would expect the slope of changing FF in the morning to be directly proportional to the extent of the difference in current FF from the idealized FF- if the syllable starts at a particularly low FF one morning we would expect the rise in FF that day to be particularly strong. Conversely, on days when the FF was higher than average (which happens less commonly but can occur) we would expect the trend of FF to have a shallower positive, or even negative slope. Indeed, this is exactly what we find; when FF is higher than average, it tends to drop, not rise, in the morning. The magnitude of FF slope in the morning is significantly associated with the difference from average daily FF (Figure 6). Factors other than error correction could also contribute to or mediate the daily oscillation in FF. In fact, because the evening fall in FF appears to involve continued change away from average, it seems likely that error correction is stronger in the morning and other factors dominate in the evening. In this regard, it might be helpful to examine whether a daily rhythm occurs in other vocalizations such as calls. Manipulating singing behavior could also probe for an effect of practice on performance.

### Daily Oscillations during Learning

One form of daily patterning to song production has been reported before; during the juvenile learning period, songs performed immediately after waking showed greater acoustic difference from the tutor song than those produced later in the day [36]. This effect was hypothesized to be a negative byproduct of “offline” learning, perhaps somehow involving the consolidation of memory during sleep, and no similar effect was reported in adults. The analysis we have done here is more focused, dealing with only FF and amplitude of individual syllables. The authors in that study do not report an evening fall in song similarity as might be expected if there were a common mechanism driving the changes seen in that study and this one, suggesting differing underlying phenomena. Nonetheless it will be interesting to see to what extent the oscillation we observe here is present in juveniles towards the end of the song learning process, although the increased variability in juvenile song may obscure the daily oscillations we report here.

## Conclusions

The FF of harmonic syllables rises across the morning, reaching a peak near mid-day, and then falls again in the late afternoon until sleep. Amplitude follows a similar pattern, albeit less clearly, likely due to difficulties in measuring amplitude accurately. These oscillations do not require normal levels of serotonin, as animals with serotonergic lesions maintained daily oscillations in both FF and amplitude. We hypothesize that these oscillations are driven by underlying physiological factors, possibly including body temperature or melatonin, which are likely shared with other vertebrates, or at least homeotherms. Song production in zebra finches is a valuable model system of complex learned behavior because of the ease of gathering strong behavioral data and the tractability of the underlying neural circuitry. The presently discovered daily oscillation promises to reveal new insights into how time of day may influence our ability to accomplish a variety of complex learned motor skills.

## References

[1] Carrier J, Monk TH (2000) Circadian rhythms of performance: new trends. Chronobiol Int 17: 719–732. doi:10.1081/CBI-100102108. PubMed: 11128289. 11128289

[2] Gates A (1916) Variation in efficiency during the day, together with practise effects, sex differences, and correlations. Berkeley: Univ Calif Press in Psychology 2(1):1-156.

[3] Kleitman N, Jackson DP (1950) Body temperature and performance under different routines. J Appl Physiol 3: 309–328. PubMed: 14794593. 14794593 10.1152/jappl.1950.3.6.309

[4] Reilly T, Walsh TJ (1981) Physiological, psychological and performance measures during an endurance record for 5-a-side soccer play. Br J Sports Med 15: 122–128. doi:10.1136/bjsm.15.2.122. PubMed: 7272654. 7272654 PMC1858731

[5] Atkinson G, Speirs L (1998) Diurnal variation in tennis service. Percept Mot Skills 86: 1335–1338. doi:10.2466/pms.1998.86.3c.1335. PubMed: 9700810. 9700810

[6] Tchernichovski O, Mitra PP, Lints T, Nottebohm F (2001) Dynamics of the vocal imitation process: how a zebra finch learns its song. Science 291: 2564–2569. doi:10.1126/science.1058522. PubMed: 11283361. 11283361

[7] Zeigler P, Marler P (2004) Behavioral Neurobiology of Birdsong. Annals of the New York Academy of Sciences 1016: 1–xvii. 10.1196/annals.1298.029. 15313767

[8] Amador A, Margoliash D (2013) A mechanism for frequency modulation in songbirds shared with humans. J Neurosci 33: 11136-11144. doi:10.1523/JNEUROSCI.5906-12.2013. PubMed: 23825417. 23825417 PMC3718373

[9] Monti JM (2010) Serotonin 5-HT(2A) receptor antagonists in the treatment of insomnia: present status and future prospects. Drugs of Today (Barcelona, Spain : 1998) 46: 183–193. PubMed: 20467592. 10.1358/dot.2010.46.3.1437247 20467592

[10] Wood WE, Roseberry TK, Perkel DJ (2013) HTR2 Receptors in a Songbird Premotor Cortical-Like Area Modulate Spectral Characteristics of Zebra Finch Song. J Neurosci 33: 2908–2915. doi:10.1523/JNEUROSCI.4291-12.2013. PubMed: 23407949. 23407949 PMC3711768

[11] Tchernichovski O, Nottebohm F, Ho CE, Pesaran B, Mitra PP (2000) A procedure for an automated measurement of song similarity. Animal Behaviour 59: 1167–1176. doi:10.1006/anbe.1999.1416. PubMed: 10877896. 10877896

[12] Leblois A, Wendel BJ, Perkel DJ (2010) Striatal dopamine modulates basal ganglia output and regulates social context-dependent behavioral variability through D1 receptors. J Neurosci 30: 5730–5743. doi:10.1523/JNEUROSCI.5974-09.2010. PubMed: 20410125. 20410125 PMC2866011

[13] Wu W, Thompson JA, Bertram R, Johnson F (2008) A statistical method for quantifying songbird phonology and syntax. J Neuroscience Methods 174: 147–154. doi:10.1016/j.jneumeth.2008.06.033. PMC2569874 18674560

[14] Tumer EC, Brainard MS (2007) Performance variability enables adaptive plasticity of “crystallized” adult birdsong. Nature 450: 1240–1244. doi:10.1038/nature06390. PubMed: 18097411. 18097411

[15] Neumaier JF, Szot P, Peskind ER, Dorsa DM, Hamblin MW (1996) Serotonergic lesioning differentially affects presynaptic and postsynaptic 5-HT1B receptor mRNA levels in rat brain. Brain Res 722: 50–58. doi:10.1016/0006-8993(96)00178-3. PubMed: 8813349. 8813349

[16] Goller F, Cooper BG (2004) Peripheral motor dynamics of song production in the zebra finch. Ann N Y Acad Sci 1016: 130-152. doi:10.1196/annals.1298.009. PubMed: 15313773. 15313773

[17] Zollinger SA, Podos J, Nemeth E, Goller F, Brumm H (2012). Animal Behaviour 84: e1-e9. doi:10.1016/j.anbehav.2012.06.013.

[18] Long MA, Fee MS (2008) Using temperature to analyse temporal dynamics in the songbird motor pathway. Nature 456: 189–194. doi:10.1038/nature07448. PubMed: 19005546. 19005546 PMC2723166

[19] Kao MH, Brainard MS (2006) Lesions of an avian basal ganglia circuit prevent context-dependent changes to song variability. J Neurophysiol 96: 1441–1455. doi:10.1152/jn.01138.2005. PubMed: 16723412. 16723412

[20] Sossinka R, Bohner J (1980) Song Types in the Zebra Finch Poephila guttata castanotis1. Zeitschrift für Tierpsychologie 53: 123–132.

[21] Glaze CM, Troyer TW (2006) Temporal structure in zebra finch song: implications for motor coding. J Neurosci 26: 991–1005. doi:10.1523/JNEUROSCI.3387-05.2006. PubMed: 16421319. 16421319 PMC6675377

[22] Aronov D, Fee MS (2012) Natural changes in brain temperature underlie variations in song tempo during a mating behavior. PLOS ONE 7: e47856. doi:10.1371/journal.pone.0047856. PubMed: 23112858. 23112858 PMC3480430

[23] Gahr M, Kosar E (1996) Identification, distribution, and developmental changes of a melatonin binding site in the song control system of the zebra finch. J Comp Neurol 367: 308–318. doi:10.1002/(SICI)1096-9861(19960401)367:2. PubMed: 8708012. 8708012

[24] Jansen R, Metzdorf R, Van der Roest M, Fusani L, Ter Maat A et al. (2005) Melatonin affects the temporal organization of the song of the zebra finch. FASEB J 19: 848–850. PubMed: 15746187. 15746187 10.1096/fj.04-2874fje

[25] Derégnaucourt S, Saar S, Gahr M (2012) Melatonin affects the temporal pattern of vocal signatures in birds. J Pineal Res 53: 245-258. doi:10.1111/j.1600-079X.2012.00993.x. PubMed: 22506964. 22506964

[26] Sober SJ, Wohlgemuth MJ, Brainard MS (2008) Central contributions to acoustic variation in birdsong. J Neurosci 28: 10370–10379. doi:10.1523/JNEUROSCI.2448-08.2008. PubMed: 18842896. 18842896 PMC2613831

[27] Wood WE, Lovell PV, Mello CV, Perkel DJ (2011) Serotonin, via HTR2 Receptors, Excites Neurons in a Cortical-like Premotor Nucleus Necessary for Song Learning and Production. J Neurosci 31: 13808–13815. doi:10.1523/JNEUROSCI.2281-11.2011. PubMed: 21957243. 21957243 PMC3220194

[28] Morin LP (1999) Serotonin and the regulation of mammalian circadian rhythmicity. Ann Med 31: 12–33. doi:10.3109/07853899909019259. PubMed: 10219711. 10219711

[29] Compan V, Segu L, Buhot MC, Daszuta A (1998) Selective increases in serotonin 5-HT1B/1D and 5-HT2A/2C binding sites in adult rat basal ganglia following lesions of serotonergic neurons. Brain Res 793: 103–111. doi:10.1016/S0006-8993(98)00168-1. PubMed: 9630549. 9630549

[30] Morin LP, Blanchard J (1991) Depletion of brain serotonin by 5,7-DHT modifies hamster circadian rhythm response to light. Brain Res 566: 173–185. doi:10.1016/0006-8993(91)91696-X. PubMed: 1814534. 1814534

[31] Mistlberger RE, Bossert JM, Holmes MM, Marchant EG (1998) Serotonin and feedback effects of behavioral activity on circadian rhythms in mice. Behav Brain Res 96: 93–99. doi:10.1016/S0166-4328(98)00007-2. PubMed: 9821546. 9821546

[32] Paulus EV, Mintz EM (2012) Developmental disruption of the serotonin system alters circadian rhythms. Physiol Behav 105: 257–263. PubMed: 21907225. 21907225 10.1016/j.physbeh.2011.08.032

[33] Nordeen KW, Nordeen EJ (1992) Auditory feedback is necessary for the maintenance of stereotyped song in adult zebra finches. Behav Neural Biol 57: 58–66. doi:10.1016/0163-1047(92)90757-U. PubMed: 1567334. 1567334

[34] Sober SJ, Brainard MS (2009) Adult birdsong is actively maintained by error correction. Nat Neurosci 12: 927–931. doi:10.1038/nn.2336. PubMed: 19525945. 19525945 PMC2701972

[35] Tumer EC, Brainard MS (2007) Performance variability enables adaptive plasticity of “crystallized” adult birdsong. Nature 450: 1240–1244. doi:10.1038/nature06390. PubMed: 18097411. 18097411

[36] Derégnaucourt S, Mitra PP, Fehér O, Pytte C, Tchernichovski O (2005) How sleep affects the developmental learning of bird song. Nature 433: 710–716. doi:10.1038/nature03275. PubMed: 15716944. 15716944

